# Bioengineering functional smooth muscle with spontaneous rhythmic contraction *in vitro*

**DOI:** 10.1038/s41598-018-31992-4

**Published:** 2018-09-10

**Authors:** Masae Kobayashi, Hassan A. Khalil, Nan Ye Lei, Qianqian Wang, Ke Wang, Benjamin M. Wu, James C. Y. Dunn

**Affiliations:** 1 Department of Bioengineering, Henry Samueli School of Engineering, University of California, Los Angeles, Los Angeles, CA 90095 USA; 2 Department of Surgery, David Geffen School of Medicine at UCLA, University of California, Los Angeles, Los Angeles, CA 90095 USA; 3 Department of Computer Science, University of North Carolina Chapel Hill, North Carolina, NC 27514 USA; 4 Division of Advanced Prosthodontics & Weintraub Center for Reconstructive Biotechnology, University of California, Los Angeles, Los Angeles, CA 90095 USA; 5 Department of Surgery, Stanford University School of Medicine, Stanford, CA 94305 USA

## Abstract

Oriented smooth muscle layers in the intestine contract rhythmically due to the action of interstitial cells of Cajal (ICC) that serve as pacemakers of the intestine. Disruption of ICC networks has been reported in various intestinal motility disorders, which limit the quality and expectancy of life. A significant challenge in intestinal smooth muscle engineering is the rapid loss of function in cultured ICC and smooth muscle cells (SMC). Here we demonstrate a novel approach to maintain the function of both ICC and SMC *in vitro*. Primary intestinal SMC mixtures cultured on feeder cells seeded electrospun poly(3-caprolactone) scaffolds exhibited rhythmic contractions with directionality for over 10 weeks *in vitro*. The simplicity of this system should allow for wide usage in research on intestinal motility disorders and tissue engineering, and may prove to be a versatile platform for generating other types of functional SMC *in vitro*.

## Introduction

The intestine is responsible for digestion, nutrient and water absorption, and waste removal. An increasing number of people are suffering from various intestinal disorders which severely affect their life expectancy and quality of life^1^. According to the Crohn’s and Colitis Foundation of America, there are approximately 1.6 million Americans suffering from inflammatory bowel disease in 2014, which is a 0.2 million increase from 2008.

When the intestine fails to function, current medical treatment options have significant limitations: total parenteral nutrition leads to unacceptably high morbidity and mortality rates and transplantation faces donor scarcity, organ rejection, and life-long immunosuppression^2^. Therefore, engineering functional intestinal tissue raises hope as an alternative therapeutic strategy for patients with intestinal failure. Previous studies have been done to regenerate the intestinal epithelium^3,4^ and many types of muscle, including vascular smooth muscle^5,6^, cardiac^7–10^ and skeletal muscle^5,11^. However, few have investigated the regeneration of intestinal smooth muscle^12,13^.

The aligned smooth muscle layers of the intestine move autonomously. Spontaneous electrical slow waves derived from pacemaker activity of the intestine arrange contractile patterns into phasic contractions that are the basis for peristalsis. Neural or hormonal inputs do not generate the intrinsic pacemaker activity of smooth muscles but affect the degree of coupling between pacemaker activity and contractions^14^. Interstitial cells of Cajal (ICC) are a specialized group of cells responsible for the pacemaker activity in visceral smooth muscles, generating and actively propagating the slow waves^15,16^.

Isolated intestinal SMCs may generate fast Ca^2+^ action potentials, but not the spontaneous slow waves in smooth muscles. Conversely, isolated ICC generate spontaneous electrical rhythmicity similar to the electrical activity in intact smooth muscles^17,18^. The binding of stem cell factor (SCF) to the receptor tyrosine kinase Kit induces a signaling pathway in ICC, which is critical for the normal development of ICC and rhythmic activity^19–23^. SCF stimulation of Kit is essential for ICC maintenance^22,24^, and Ca^2+^ activated Cl^−^ channels (Ano1) expressed on ICC were identified as the key conductance responsible for the pacemaker activity^18^. However, ICC comprise less than 10% of the cells within intestinal smooth muscle, and isolated ICC are difficult to maintain in cell culture. ICC tend to lose their characteristic morphologies and Kit immunoreactivity after enzymatic digestion during the cell isolation procedure. Although ICC can grow and develop networks that produce electrical rhythmicity in cell culture, those dispersed ICC undergo extensive phenotypic changes within a few days, including the apparent loss of the ion channels responsible for the slow wave activity^14,18,25,26^.

STO cells are embryonic fibroblasts commonly used for embryonic stem cell and primordial germ cell cultures^27–29^ due to its potent growth factors and cytokines production ability including SCF^30^ essential for ICC. Using this STO feeder cell system, we demonstrate a novel protocol where both purified ICC and isolated intestinal smooth muscle cell (ISMC) mixtures can be cultured for weeks *in vitro* with intact pacemaker ability and rhythmic contractions. Disruption of ICC networks and the loss of SMC maturity in intestinal muscularis have been reported in a variety of diseases resulting in intestinal motility disorders leading to pseudo-obstruction, Hirschsprung’s disease, inflammatory bowel diseases, and slow transit constipation^31–34^. Therefore, this system provides not only crucial progress in intestinal smooth muscle engineering, but also an *in vitro* platform to investigate cellular phenotypes and mechanisms associated with different intestinal disorders, to screen drugs that may alter motility, and to identify the biomarkers for early diagnosis and clinical stratification.

Moreover, this system may assist in maintaining and enhancing the maturity of SMCs from other vesicular organs, including the bladder, uterus, and vasculatures, due to their similar phenotypes^35,36^.

## Results

### ICC proliferation *in vitro*

**Figure 1:**
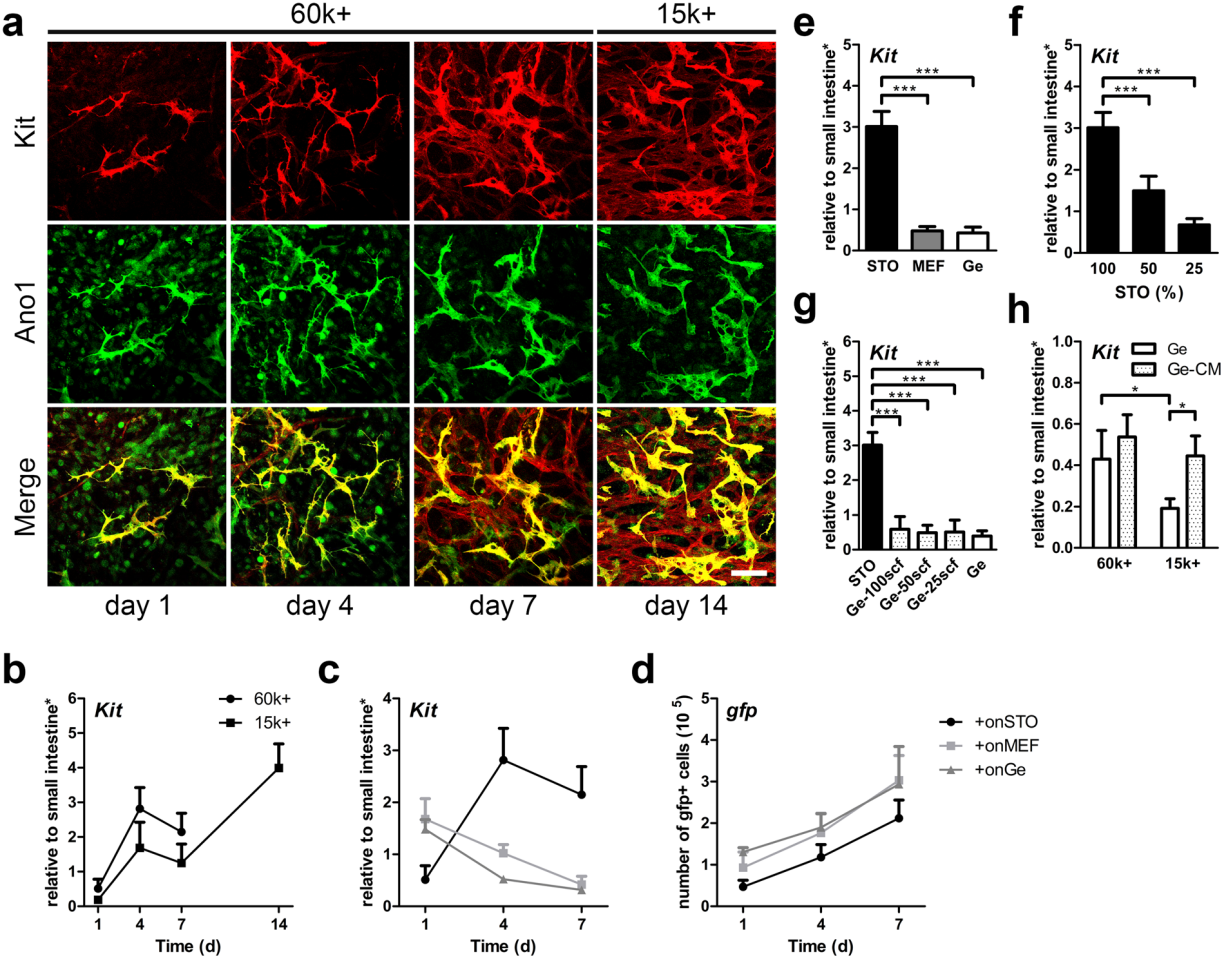
Maintenance of MACS+ cells cultured on STO cells. (**a**) Confocal images of ICC markers, Kit (red), Ano1 (green), and co-localization (yellow). 60 k MACS+ and 15 k MACS+ cells were cultured for 7 days and 14 days respectively. Scale bar, 100 µm. (**b**) MACS+ cells were analyzed for Kit mRNA expression (**b**–**d**: day 1, 4, 7 *n* = *4*; day 14 *n* = *2*). (**c,d**) GFP + MACS+ cells were seeded on STO cells, MEF, and Ge, cultured, and analyzed for mRNA expression of *Kit* (**c**) and *gfp* DNA (**d**). STO cells and MEF do not express *Kit/gfp*. (**e**) mRNA expression of *Kit* for MACS+ cells cultured on STO cells, MEF and Ge for 7 days. (**f**) mRNA expression of *Kit* for MACS+ cells cultured on different STO seeding densities for 7 days, where 100% is 100 k (100%, *n* = *5*; 50%, *n* = *4;* 25%, *n* = *2*). (**g**) mRNA expression of *Kit* for MACS+ cells cultured on Ge for 7 days in media supplemented with 25, 50, or 100 ng/ml of soluble scf (*n* = *2*). STO and Ge were controls (*n* = *5*). (**h**) mRNA expression of *Kit* for 60 k or 15 k MACS+ cells cultured on Ge for 7 days supplemented with conditioned media from STO (Ge-CM), where Ge was the control (*n* = *4*). STO = Mouse Embryonic Fibroblfast (Sandos Inbred Mouse, SIM). MEF = Mouse Embryonic Fibroblast (C57BL/6). Ge = gelatin coating. Feeder cells (STO, MEF) were mitomycin C treated. *Samples were normalized to de-epithelialized intestine. Error bars, s.d. ***P < 0.0001, *P < 0.05.

To enrich the ICC population in culture, immunomagnetic sorting was performed on primary intestinal smooth muscle cell mixture (ISMC Mix, derived from C57BL/6 mouse) that were first cultured on gelatin for 3 days. The resultant MACS+ cells (MACS purified ICC population using antibody to Kit) proliferated on mitomycin-treated STO cells that expressed stem cell factor (SCF). The MACS+ cells exhibited ICC morphology and expressed Kit and Ano1 for up to two weeks in culture (Fig. 1a,b). When a 60 k per well cell seeding density was used, MACS+ cells spread on STO cells in 48-well plates and became confluent in one week. A lower seeding density was tested to achieve more cell expansion. MACS+ cells with seeding density of 15 k proliferated on STO cells and formed a confluent layer in two week (Fig. 1a,b).

Since STO cells are embryonic fibroblasts commonly used for embryonic stem cell culture, embryonic fibroblasts derived from the same mouse strain as ISMC Mix (C57BL/6), MEF, were also tested with MACS+ cell culture, with gelatin-coated wells serving as controls. STO cells supported MACS+ cell growth better than gelatin coating and MEF cells (Fig. 1c–e). Although MACS+ cells proliferated on all three conditions (Fig. 1d), cells maintained the ICC marker Kit significantly better on STO cells (3.01 ± 0.347) than on MEF (0.442 ± 0.133) or gelatin(0.395 ± 0.150) (Fig. 1c,e and Supplementary Fig. 1). Although with the same 100 k cell seeding density, more STO cells than MEF were attached because the size of STO cells (1.00 ± 0.213) is smaller than MEF (5.05 ± 3.03) (Supplementary Fig. 2a,c). STO cells expressed significantly higher *kitl* mRNA level at day 0 (0.24 ± 0.026 vs. 0.042 ± 0.002) and after 7 days (1.56 ± 0.121 vs. 1.09 ± 0.069) in culture than MEF cells (Supplementary Fig. 2d,e). STO cells also expressed more SCF protein at day 7 than MEF cells (Supplementary Fig. 2b). The difference in SCF expression may be responsible for the difference in ICC survival. This is further supported by varying STO cell density, which demonstrated a density-dependent proliferation of MACS+ cells^30^ (Fig. 1f).

Exogenously added SCF, however, was insufficient to support ICC survival. Concentrations up to 100 ng/ml of free SCF added to the culture medium failed to preserve ICC phenotype (Fig. 1g). There may be additional factors secreted by STO cells that are beneficial for ICC growth. When STO conditioned media (CM) was mixed into the culture medium (1:1 ratio) for MACS+ cell culture, CM provided a cell density dependent benefit to MACS+ cells. CM provided significant improvement in MACS +cell growth on gelatin only at a lower seeding density (0.445 ± 0.097 vs. 0.191 ± 0.047) compared to control (Fig. 1h). Providing CM to a low seeding density (0.445 ± 0.097), MAC+ cells expressed Kit to a level comparable to MACS+ cells seeded at higher density without CM (0.429 ± 0.140) (Fig. 1h).

**Figure 2:**
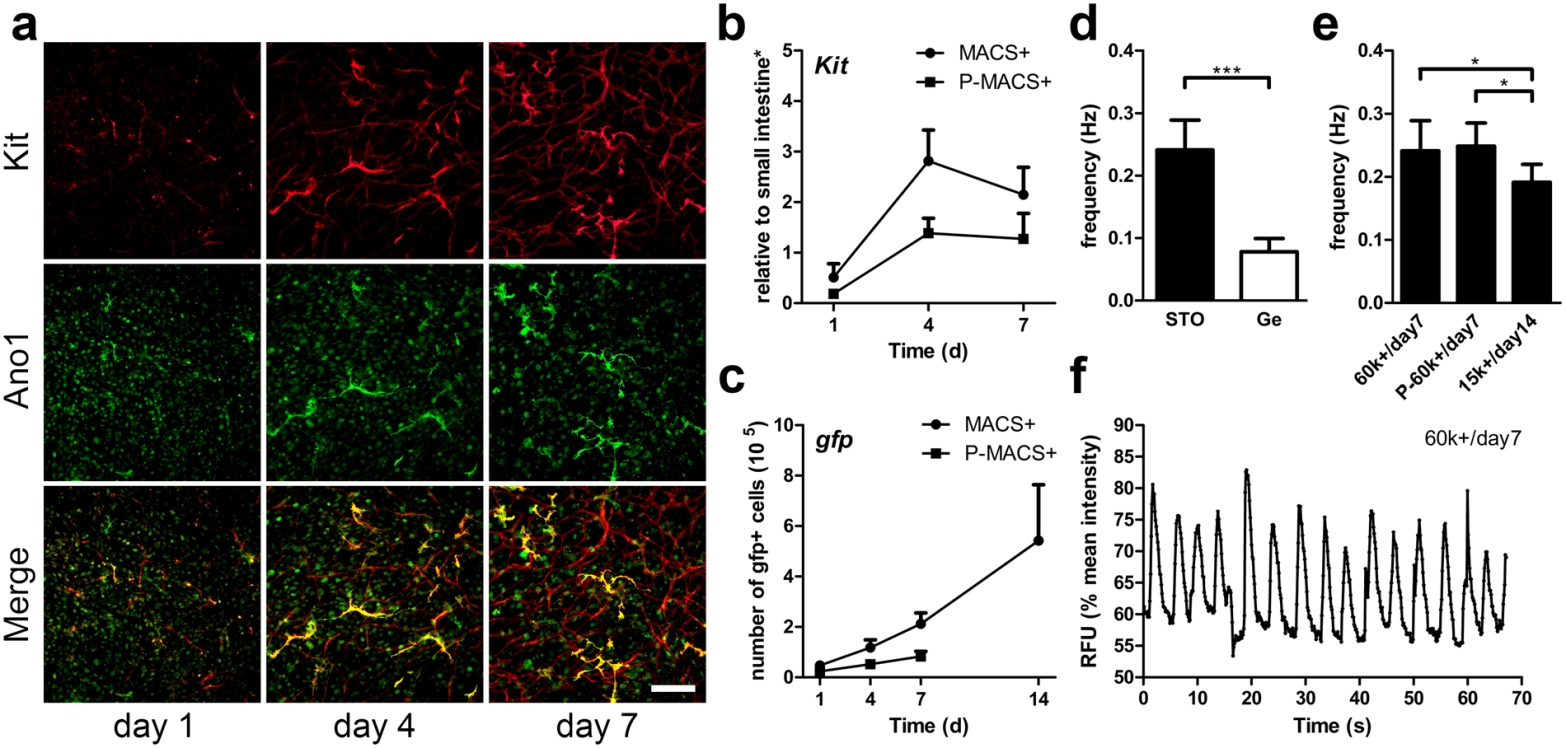
Maintenance of passaged MACS+ cells on STO cells and rhythmic pacemaker activity of cultured ICC (MACS+ and passaged MACS+ cells). 60 k sorted cells were cultured for 7 days unless otherwise noted. (**a**) Immunofluorescence of passaged MACS+ (P-MACS+) cells with ICC markers, Kit (red) and Ano1 (green) and with co-localization (yellow). MACS+ cells were cultured on STO cells for 7 days and were passaged and sorted with MACS (P-MACS+). P-MACS+ cells were subsequently cultured on STO cells. Scale bar, 200 µm. (**b,c**) Growth comparison of GFP + MACS+ and P-MACS+ cells with mRNA expression of *Kit* (**b**) and DNA expression of *gfp* (**c**). (**b,c**: day 1, 4, 7 *n* = *4*; day 14 *n* = *2*). (**d**–**f**) Oscillations in intracellular Ca^2+^ concentration demonstrated the rhythmic pacemaker activity in MACS+ and P-MACS+ ICC cultures and their frequency were measured. (**d**) Ca^2+^ oscillation frequency of MACS+ cells cultured on STO cells or Ge at day 7 (*n* ≥ *5*). (**e**) Ca^2+^ oscillation frequency of 60 k MACS+ and 60 k P-MACS+ cells cultured on STO cells at day 7, and the frequency of 15 k MACS+ cells cultured on STO cells at day 14 (*n* ≥ *5*). (**f**) Representative time-course change in fluorescence intensity due to Ca^2+^ oscillation of ICC in the culture of MACS+ cells on STO cells at day 7 (Supplementary Video 1). *Samples were normalized to de-epithelialized intestine. Error bars, s.d. ***P < 0.0001, *P < 0.05.

The cultured MACS+ cells were passaged by performing an additional sorting on MACS+ cells growing on STO cells. Such passaged MACS+ cells (P-MACS+ cells) were seeded again on STO cells. Although the growth rate was slower, P-MACS+ cells also proliferated on STO cells and exhibited ICC morphology and expressed Kit and Ano1 (Fig. 2a–c).

### Rhythmic pacemaker activity of cultured ICC (MACS+ and P-MACS+ cells)

To determine whether cells cocultured with STO cells were functional, we examined the intracellular Ca^2+^ oscillation frequency in MACS+ and P-MACS+ cells cultured on STO cells or gelatin at different density. With a higher seeding density (60 k), the MACS+ cells on STO cells exhibited a rhythmic Ca^2+^ oscillation frequency that was significantly higher (0.241 ± 0.048 Hz vs. 0.078 ± 0.021 Hz) than MACS+ cells cultured on gelatin (Fig. 2d). STO cells also supported P-MACS+ cells seeded at a higher density, and these cells exhibited a rhythmic Ca^2+^ oscillation frequency similar (0.249 ± 0.037 Hz vs. 0.241 ± 0.048 Hz) to MACS+ cells before passaging (Fig. 2e). Moreover, it’s easier to maintain and restore the functionality of MACS+ cells seeded at a higher density. With a lower seeding density (15 k), it was still possible to recover the functionality of MACS+ cells, but it took a longer culture time (day14 vs. day7) and resulted in slightly slower frequency (0.191 ± 0.029 Hz vs. 0.241 ± 0.048 Hz) than a higher seeding density (60 k) (Fig. 2e). The number of functional cells in P-MACS+ culture was about half of that in MACS+ culture due to a slower growth rate (Fig. 2a–c). A higher seeding density and lower passage number were beneficial for cells to form a network sooner, which lead to an earlier establishment of coordinated pacemaker activity in culture (Fig. 2e). Moreover, MACS+ cells cultured on STO cells demonstrated stable, periodic Ca^2+^ oscillation over time (Fig. 2f and Supplementary Video 1) and its average frequency (0.241 ± 0.048 Hz) was in the range of previous studies^18,21,37–39^. Our results suggest that the STO culture system facilitates proliferation of not only MACS+ cells but also P-MACS+ cells over time while preserving their pacemaker activities.

### Application of MACS+ cells cultured on STO cells

**Figure 3:**
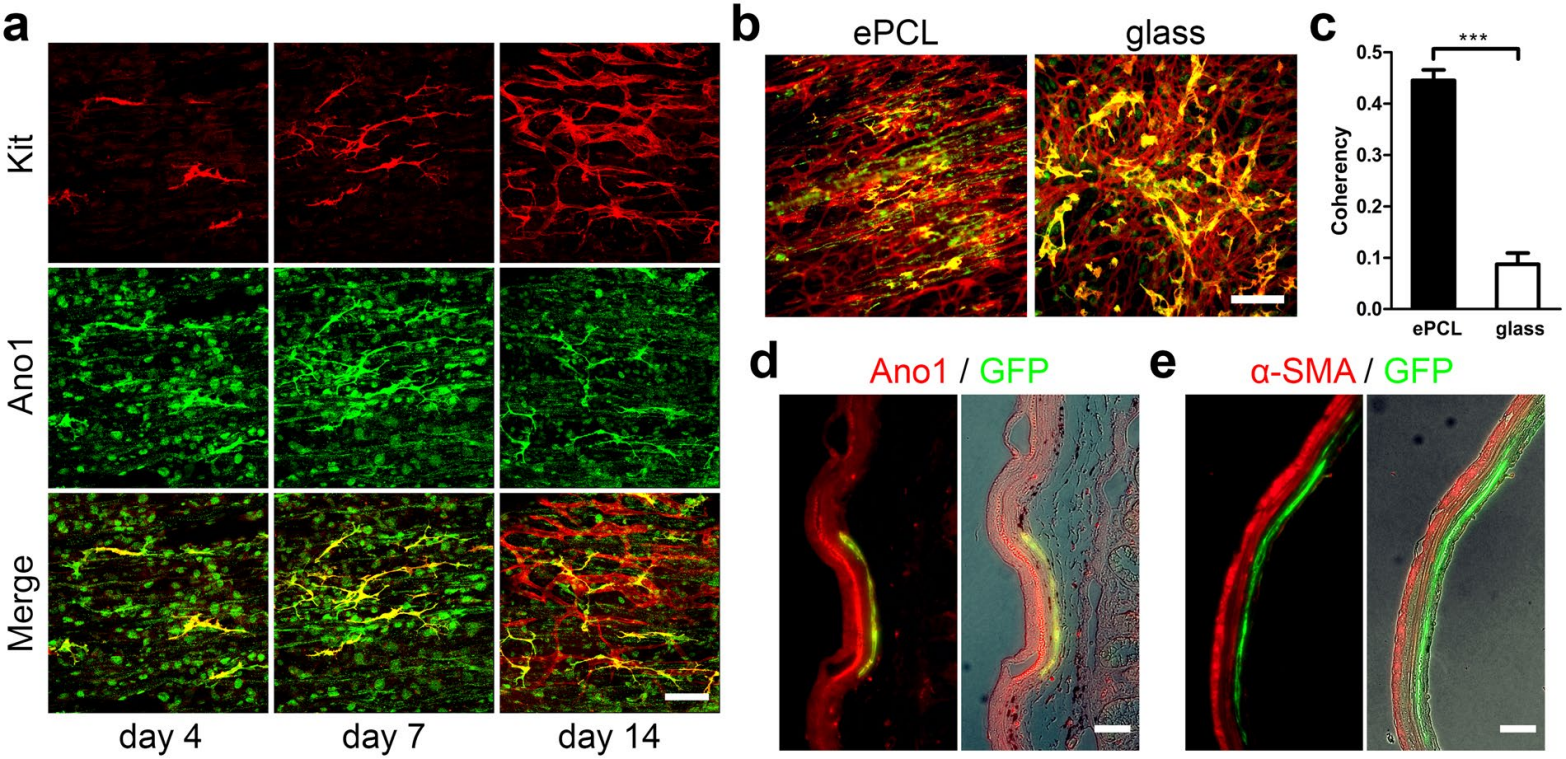
Application of MACS+ cells cultured on STO cells. (**a**) Confocal images of ICC markers, Kit (red), Ano1 (green), and co-localization (yellow). 15 k MACS+ cells were cultured on STO-seeded ePCL scaffold for 14 days. Scale bar, 100 µm. (**b,c**) Quantification of Kit alignment expressed by 15 k MACS+ cells cultured on STO-seeded ePCL and glass for 14 days. (**b**) Immunofluorescence of ICC markers, Kit (red) and Ano1 (green) and with co-localization (yellow). Scale bar, 200 µm. (**c**) Coherency analysis of Kit expression of MACS+ cells cultured on ePCL(aligned) and glass (random), where higher coherency means greater cell alignment (*n* = *5*). (**d,e**) GFP + MACS+ cells were cultured on STO cells for 4 days and purified using MACS before colonoscopic injection into the submucosa of the rectum of C57BL/6 mice. A permanent carbon ink was mixed into the cell suspension to mark injection sites. The injected rectums were retrieved after 7 days, and were immunostained for Ano1 (**d**, red), α-SMA (**e**, red) and GFP (**d,e**, green). GFP co-localized with Ano1 (**d**, yellow) but not with α-SMA (**e**). Merged immunofluorescence images (left) were further merged with phase contrast images to show the area of injection indicated by black ink (right). Scale bar, 100 µm. Insets, higher-magnification images of boxed regions. Scale bar, 10 µm. Error bars, s.d. ***P < 0.0001.

ICC residing in the intestinal smooth muscle tend to align along the smooth muscle orientation^40–43^ (Supplementary Fig. 3a). In order to induce the proper alignment, MACS+ cells were cultured on STO-seeded electrospun poly(3-caprolactone) (ePCL) scaffolds^40^ (Supplementary Fig. 3b**)**. MACS+ cells proliferated and aligned along the ePCL fiber orientation after 2 weeks (Fig. 3a). Coherency analysis of Kit expression confirmed that MACS+ cells cultured on ePCL scaffolds (aligned) were statistically more aligned (0.445 ± 0.021 vs. 0.087 ± 0.022) than those cultured on glass (random orientation) controls (Fig. 3b,c). Further, we demonstrated that MACS+ cells (GFP+) survived for at least a week *in vivo*, following colonoscopic injection into the rectal submucosa of C57BL/6 mice. Injected MACS+ cells, identified by GFP, co-localized with Ano1 expressed by ICC but not with α-SMA (Fig. 3d,e). Moreover, MACS+ cells were distributed around the injection site of the submucosa as indicated by carbon ink and demonstrated alignment along the smooth muscle layer, accompanied by native ICC and SMCs (Fig. 3d,e). Minimal inflammation was noted at the site of cell injection. These results demonstrate that the *in vitro* expanded ICC not only can be align by using scaffolds but also can survive *in vivo* through colonoscopic injections.

### ISMC Mix and MOVAS cells maintenance on STO cells

**Figure 4:**
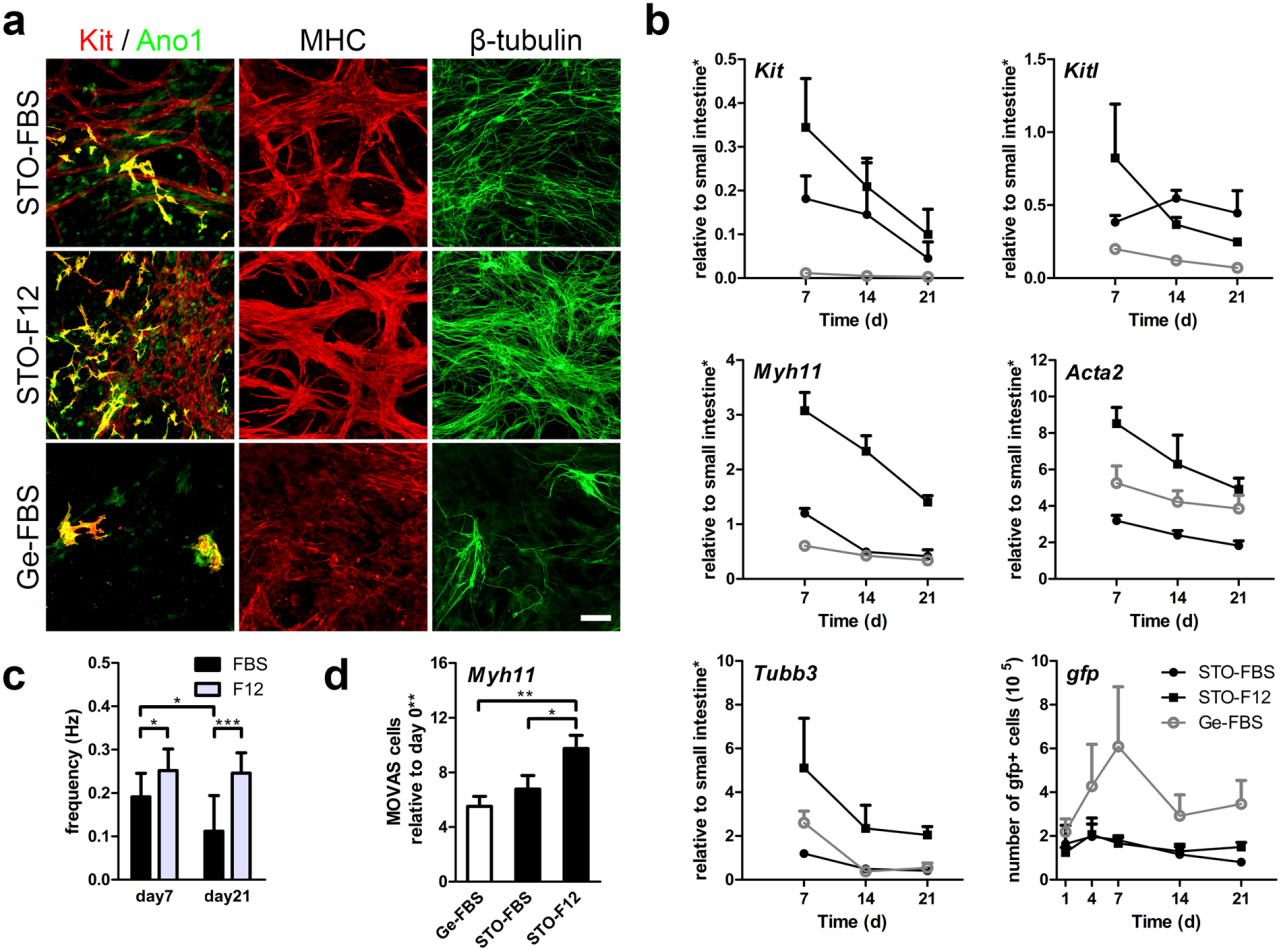
Maintenance of intestinal smooth muscle cell mixture (ISMC Mix) and vascular aortic smooth muscle cell line (MOVAS cells) cultured on STO cells *in vitro*. Non-sorted cells from enzymatically digested intestinal muscle strips were used as ISMC Mix. All ISMC Mix and MOVAS cells were cultured separately in FBS medium for the first 4 days followed by FBS or F12 medium. (**a**–**c**) 100 k ISMC Mix were cultured on STO cells or Ge with FBS or F12 medium for up to 3 weeks. (**a**) Immunofluorescence of ISMC Mix with ICC markers showing co-localization (yellow) of Kit (red) and Ano1 (green), SMCs marker MHC (red), and neuronal marker β-tubulin (green) at day 7. Scale bar, 100 µm. (**b**) GFP + ISMC Mix were analyzed for mRNA expression (*Kit, Myh11, Tubb*3*, Kitl*, and *Acta2*) and *gfp* DNA (*n* = *3*). (**c**) Spontaneous contraction demonstrated the functionality of ISMC Mix cultured on STO cells with FBS or F12 medium (Supplementary Video 2 and 3) and frequency was measured (*n* ≥ *5*). (**d**) MOVAS cells were analyzed for mRNA expression (*Myh11*) at day 7 (*n* = *3*; triplicate samples). FBS = 15% FBS in DMEM. F12 = advanced DMEM/F12. *Samples were normalized to de-epithelialized intestine. **Samples were normalized to 100 k cells from day 0. Error bars, s.d. ***P < 0.0001, *P < 0.05.

To test whether our approach of culturing MACS+ cells with spontaneous Ca^2+^ oscillation could support periodic contractions of SMCs, ISMC Mix was cultured on STO cells for three weeks. At day 7, ISMC Mix cultured on STO cells in F12 medium expressed the highest level of ICC markers (Kit and Ano1 co-localization), SMCs marker (MHC) and neuronal marker (β-tubulin), followed by ISMC Mix cultured on STO cells in FBS medium and ISMC Mix cultured on gelatin in FBS medium (Fig. 4a). Comparable to the trends observed in immunostaining, ISMC Mix on STO cells cultured in F12 medium showed the greatest expression of *Kit, Myh11, Tubb3, Kitl*, and *Acta2* up to three weeks, with the exception that cells cultured on STO cells in FBS medium had a slightly higher expression of *Kitl* at weeks two and three (Fig. 4b). Cells cultured on STO cells had higher expression of *Kit and Kitl* (STO-FBS vs. Ge-FBS) while F12 medium induced higher expression of *Myh11, Tubb3*, and *Acta2* (STO-F12 vs. STO-FBS) (Fig. 4b). Cultures with STO cells and F12 medium demonstrated the greatest expression of *Myh11* over time (Fig. 4b). ISMC Mix cultured on gelatin had a higher proliferation but lower expression of other markers compared to ISMC Mix cultured on STO cells with FBS or F12 medium (Fig. 4b). We also cultured a vascular aortic smooth muscle cell line (MOVAS cells) under the same conditions as ISMC Mix to study the effects of our system on non-intestinal SMCs. MOVAS cells followed a similar trend as ISMC Mix; they were enriched in mature SMC marker, *Myh11*, on day 7, with the best condition being F12 medium on STO cells (9.75 ± 0.969) and the worst being on gelatin (5.51 ± 0.735) (Fig. 4d). This finding suggests that the beneficial effects of our system on SMCs are not limited to ISMC Mix cells.

As predicted from the immunostaining and mRNA expression, ISMC Mix cultured on STO cells demonstrated periodic contractions (Fig. 4c) while cells cultured on gelatin in FBS medium did not contract (not shown). STO cells with F12 medium enabled ISMC Mix to retain faster frequency over a longer period of time (0.252 ± 0.050 vs. 0.191 ± 0.054 at day7; 0.246 ± 0.046 vs. 0.112 ± 0.082 at day21) compared to ISMC Mix cultured on STO cells in FBS medium (Fig. 4c). STO cells were shown to be effective in maintaining not only functional ICC but also functional ISMC. Overall, these results suggest that STO cells with F12 medium is the optimal condition to culture ISMC Mix with periodic contractions (Supplementary Videos 2 and 3).

### Engineering functional intestinal smooth muscle

**Figure 5:**
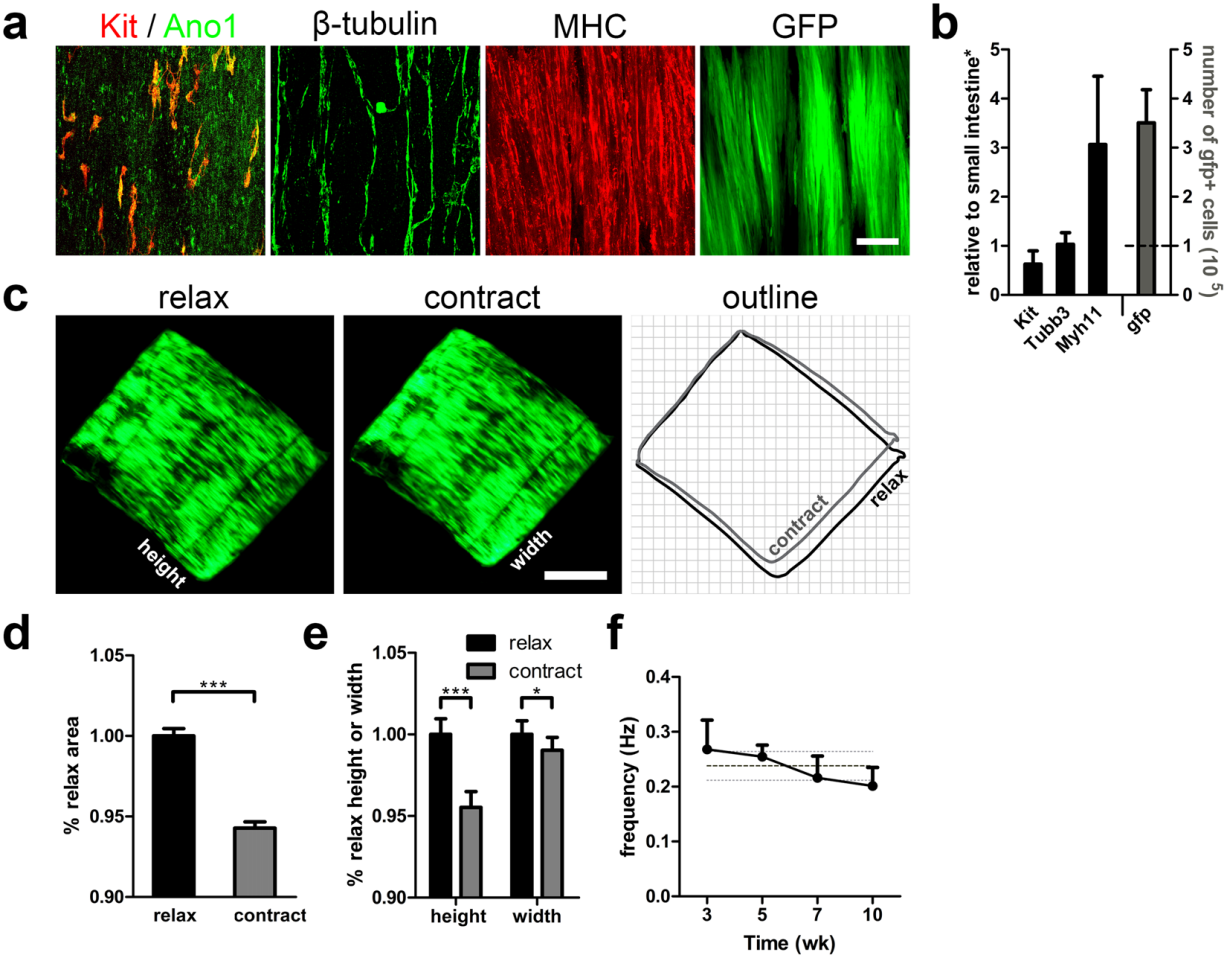
Engineering aligned intestinal smooth muscle with periodic contraction over 10 weeks *in vitro*. 100 k ISMC Mix were cultured on STO cells seeded ePCL scaffolds in FBS medium for the first 4 days before changing to F12 medium. (**a**) Confocal images of ISMC Mix with ICC markers showing co-localization (yellow) of Kit (red) and Ano1 (green), SMCs marker MHC (red), neuronal marker β-tubulin (green), and GFP (green) at 10 weeks. Scale bar, 100 µm. (**b**) GFP + ISMC Mix were analyzed for mRNA expression (*Kit, Myh11, Tubb3, Kitl*, and *Acta2*) (*n* = *5*) and *gfp* DNA (*n* = *4*). The dashed line indicates the seeding density. (**c**–**e**) Relaxed and contracted state comparison of engineered intestinal smooth muscle. (**c**) Images of GFP expression from ISMC Mix seeded ePCL scaffold were extracted from a video recording and outlined (relax, black; contract, gray) for comparison. Scale bar, 2 mm (Supplementary Video 4). (**d)** To show the degree of the periodic contraction, area of contracted ePCL scaffolds were normalized to that of relaxed ePCL scaffolds (*n* = *10*). (**e**) To show the directionality of the periodic constriction, height and width of contracted ePCL scaffolds were normalized to those of relaxed ePCL scaffolds (*n* = *10*). (**f**) Frequency of ePCL rhythmic contractions (*n* = *4*). The dark gray dashed line indicates the mean frequency over time and light gray dashed lines indicate its s.d. *Samples were normalized to de-epithelialized intestine. Error bars, s.d. ***P < 0.0001, *P < 0.05.

To create an implantable tissue construct for regenerative medicine, we engineered intestinal smooth muscle with proper alignment and functionality by culturing ISMC Mix on STO-seeded ePCL in F12 medium. ISMC Mix cultured on STO-seeded ePCL demonstrated unceasing, rhythmic contraction over 10 weeks *in vitro* (Fig. 5f). After 10 weeks of culture, the constructs were examined with immunostaining and qPCR, demonstrating expression of ICC, SMC and neuronal markers, and more than 3 fold cell growth with *gfp* DNA (Fig. 5a,b). Although STO cells played essential roles in supporting ISMC Mix culture, the effect of STO cells likely declined over time due to its mitomycin C treatment. When STO cells were seeded alone, the reduction in the cell number and viability were observed over time (Supplementary Fig. 4).

To quantify the intensity and directionality of the periodic contractions generated by ISMC Mix on ePCL scaffolds, the dimensions of the most contracted scaffold was compared to those in the most relaxed scaffold (normalized to 1). The change in area of the scaffold (0.943 ± 0.004 vs. 1.00 ± 0.005) (Fig. 5d) was mainly due to a significant change in the length of the scaffold along the direction of the fibers, labelled as the height of the ePCL scaffold (0.955 ± 0.010 vs. 1.00 ± 0.005) (Fig. 5e). Moreover, the cells cultured on ePCL scaffolds showed surface alignment along the direction of the fibers in the scaffold (Fig. 5a,c). The alignment of ISMC Mix inside the ePCL scaffolds was also quantified as we had previously described^40^. Briefly, ePCL scaffolds were cut in two orthogonal planes to expose cross section parallel or perpendicular to ePCL’s aligned fibers. Coherency analysis (MHC, GFP and β-tubulin) and circularity analysis (DAPI) were used to compare the alignment in the two cross sections, where coherency factor ranges from 0 (perfect random) to 1 (perfect alignment) indicating cell alignment while circularity factor ranges from 0 (most elongated shape) to 1 (perfect circle) indicating elongation of nuclei due to cell alignment. Our results showed that the ISMC Mix cells infiltrated inside the ePCL and arranged along the ePCL’s aligned fibers (Supplementary Fig. 5). Culturing ISMC Mix on STO-seeded ePCL produced intestinal smooth muscle constructs with proper alignment and spontaneous rhythmic contractions (Supplementary Video 4).

### ICC is essential for rhythmic contraction of ISMC Mix

**Figure 6:**
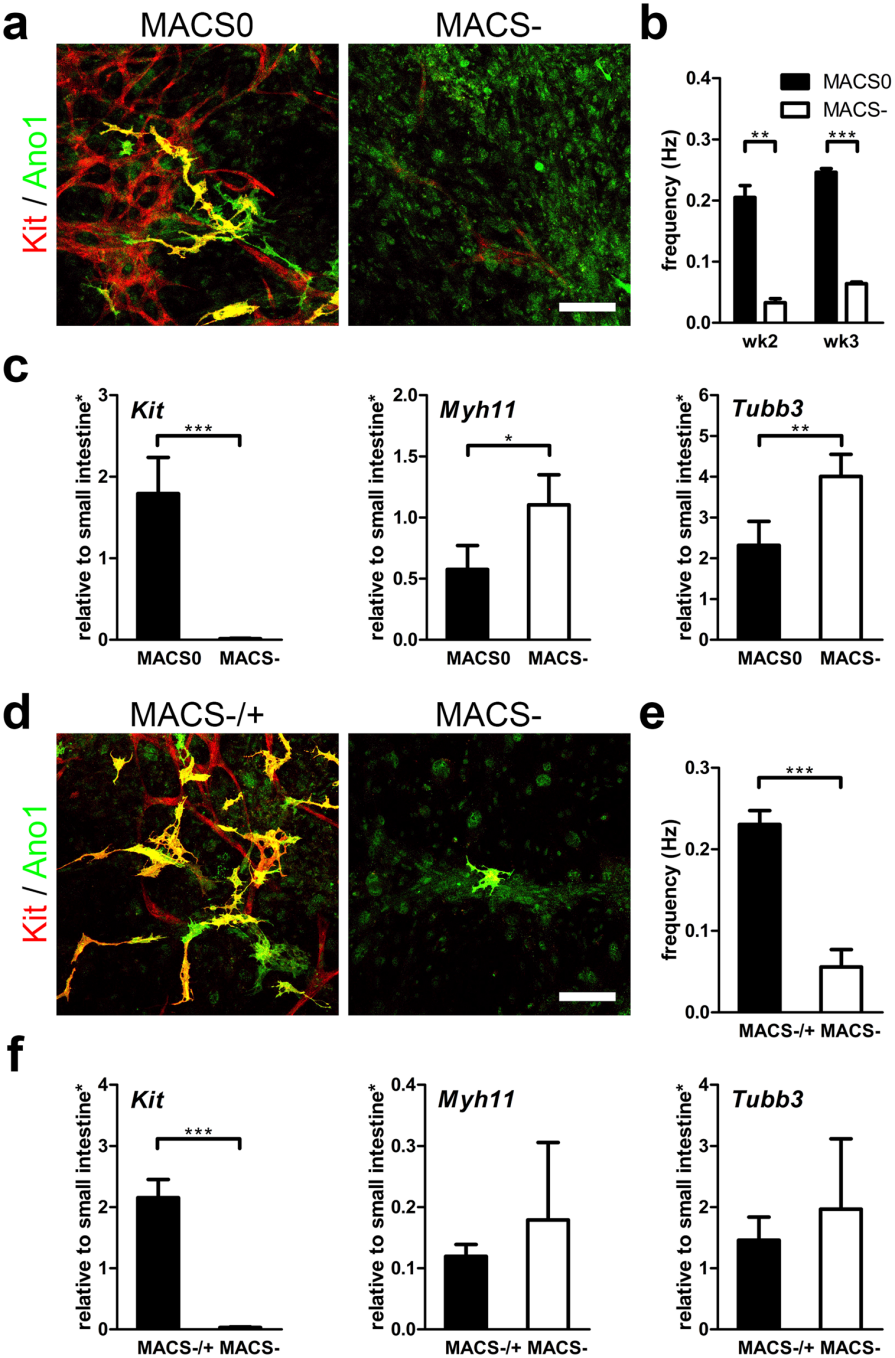
MACS+ cells’ role in cultured ISMC Mix with rhythmic contractions *in vitro*. (**a**–**c**) 100 k MACS0 (passaged ISMC Mix: mixture of MACS+ cells, SMCs and neuronal cells) or MACS− cells (ISMC Mix without MACS+ cells: mostly SMCs and neuronal cells) were cultured on STO cells for 3 weeks. Cells were seeded and cultured in FBS medium for the first 4 days before changing to F12 medium. (**d**–**f**) 100 k MACS− were seeded on STO cells for 5 weeks, with (MACS−/+) or without (MACS−) addition of 60 k MACS+ cells on day 5. Cells were cultured in FBS medium for the first 7 days before changing to F12 medium. (**a,d**) Confocal images of ICC markers, Kit (red), Ano1 (green), co-localization (yellow). Scale bar, 100 µm. (**b,e**) Frequency of cultured cells motility due to spontaneous contraction (**b**: wk2 *n* = *2*, wk3 *n* = *4*; (**e**) wk5 *n* = *4*). (**c,f**) Cultured cells were analyzed for mRNA expression (*Kit, Myh11, Tubb3*) at week 2 (**c**) or week 5 (**f**) (*n* = *4*). *Samples were normalized to de-epithelialized intestine. Error bars, s.d. ***P < 0.0001, **P < 0.01, *P < 0.05.

To confirm the role of ICC in ISMC Mix in producing spontaneous, rhythmic contractions, MACS+ cells were separated from ISMC Mix to create a MACS− cells population that contains few ICC. The MACS− cells were cultured on STO cells for 3 weeks *in vitro*, and its motility, gene and protein expression were analyzed. The comparison between passaged ISMC Mix (MACS0) and MACS− cells showed that the ICC population in MACS0 is essential for spontaneous rhythmic contractions (Fig. 6a–c). MACS− cells, without an ICC population (*Kit* 1.79 ± 0.445 vs. 0.013 ± 0.005), failed to exhibit rhythmic contractions (0.246 ± 0.006 Hz vs. 0.064 ± 0.003 Hz at week3) even though SMCs (*Myh11* 0.575 ± 0.197 vs. 1.10 ± 0.245) and neuronal cells (*Tubb3* 2.32 ± 0.586 vs. 4.01 ± 0.539) were present (Fig. 6a–c, MACS0 vs. MACS−). We also performed a rescue experiment where MACS+ cells were added back to MACS− cells 5 days after they were cultured on STO cells (MACS−/+). The added MACS+ cells restored the rhythmic contractions of the MACS− cells cultured on STO cells in 5 weeks (0.230 ± 0.017 Hz vs. 0.056 ± 0.021; *Kit* 2.15 ± 0.297 vs. 0.032 ± 0.010; *Myh11* 0.119 ± 0.019 vs. 0.179 ± 0.127; *Tubb3* 1.46 ± 0.378 vs. 1.97 ± 1.15) (Fig. 6d–f, MACS−/+ vs. MACS−). This experiment confirms that ICC play an essential role in producing the spontaneous rhythmic contractions of cultured ISMC.

## Discussion

This study demonstrates that STO cells can support ICC in culture and maintains the spontaneous intracellular Ca^2+^ oscillation frequency within the previously published range^16,18,26,37–39,44–46^. These functional ICC pace the rhythmic contractions of SMC. It is also necessary for SMC to retain its maturity and express MHC to contract^12,13^ upon receiving pacemaker signals from functional ICC. The loss of maturity and functionality of either ICC or SMC in culture is a major challenge for intestinal smooth muscle engineering^12,13,47^. Our system described here is an approach to achieving this goal by maintaining the functionality of both ICC and SMC, as well as neuronal cells cultured *in vitro*.

This system provides an easily accessible *in vitro* model to investigate the molecular mechanisms underlying a functional smooth muscle. The combination of STO cells and F12 medium works well to produce the *in vitro* culture of the intestinal smooth muscle. While it is likely that STO cells provide signals including preferable forms of SCF^48,49^ and other factors^30^ important for ICC to survive and function, the exact mechanisms that underlie these observations will need to be investigated further. The beneficial effects provided by STO cells are important during the initial phase of the smooth muscle cell culture. It is likely that other cell types within ISMC Mix eventually take over the function of STO cells due to the mitomycin C treatment.

This novel culture method is likely to have broad applications. We demonstrated that both MACS+ cells and ISMC Mix were able to attach and align along ePCL scaffolds and that MACS+ cells cultured using our system survived *in vivo* after colonoscopic injections. Specifically, colonoscopic injections of ICC may help motility disorders including diabetic gastroparesis, pseudo-obstruction and Hirschsprung’s disease due to frequent defects in ICC networks reported in those disorders^33,34^. The culture method could also be applied to cells from other visceral organs^35^, and the cultured cells could be seeded on other biomaterials, used in other *in vivo* studies, and incorporated into microfluidic systems.

Finally, this culture system is simple and reproducible for generating a functional smooth muscle layer *in vitro*. Our STO cell system only requires standard culture medium without specialized additives. STO cells are readily available at a low cost from commercial vendors. We believe that the STO cell system has the potential to become a common method to create functional smooth muscle *in vitro*. Our data demonstrate that the system sustains the maturity of the vascular smooth muscle cells, so it may similarly serve as the platform for both basic and applied research in various other types of smooth muscle functionalities and disorders.

## Methods

### Electrospinning

11% (w/w) solution of PCL (Durect Lactel, Birmingham, AL) was made in hexafluoro-2-propanol (Acros Organics, Thermo Fisher Scientific, Waltham, MA). The solution was kept on a shaker overnight to obtain a homogenous polymer solution. The mandrel was wrapped with aluminum foil to ease the removal of the scaffold. The PCL solution was transferred to a plastic syringe fitted with an 18-gauge needle, and secured onto a syringe pump (Harvard Apparatus, Holliston, MA). The solution was infused at 2.5 ml/h onto a rotating mandrel collector with an outer diameter of 32 mm that was positioned 12–15 cm away from the needle tip. The electrical potential difference between the needle (i.e., polymer solution) and the grounded mandrel collector was produced by a high voltage power supply (Glassman High Voltage, High Bridge, NJ). Scaffolds comprised of aligned ePCL fibers were fabricated using a mandrel rotational speed of 3450 rpm and an applied voltage of 15 kV. Less-aligned, “random” ePCL fibers were produced using a mandrel rotational speed of 1725 rpm and applied voltage of 25 kV. After 0.5 ml of polymer solution had been dispensed from the syringe onto the rotating mandrel, the ePCL was carefully removed from the aluminum foil. Scaffolds were air-dried before laser cutting.

### Laser cutting

ePCL scaffolds were constructed as fiber sheets with dimensions approximately 10 × 2.5 cm and thickness of 100–150 µm, based on the mandrel used. These fiber sheets were cut into rectangular 8 × 6.5 mm scaffolds using the VERSA LASER CUTTER 2.3 (Universal Laser Systems, Scottsdale, AZ) with vector mode, 5% power, 100x speed, and 1000 pulses/inch. Scaffolds were obtained by setting the longer edge of the rectangle to be along the fiber direction. The scaffolds were cleaned with air plasma (Harrick Plasma, Ithaca, NY) at 500 mTorr, high power for 1 min and subsequently sterilized with strong UV (Dymax) for 1 min. The scaffolds were washed once with phosphate buffered saline (PBS) (Invitrogen, Carlsbad, CA) before and after coating with gelatin solution (attachment factor solution (AF); Invitrogen) at 37 °C for at least 30 min. (Note: To improve the cell seeding onto ePCL scaffolds, PDMS molds with a well size the same as an ePCL scaffold were manufactured. This will allow ePCL scaffold to fit perfectly and stay at bottom of the PDMS well so that seeded cells attached effectively on the ePCL scaffold with minimal spillage to the bottom of the well).

### Scanning Electron Microscopy (SEM)

The surface morphology of ePCL scaffolds with aligned or random fibers was assessed using a Nova NanoSEM 230 (FEI, Hillsboro, Oregon). The scaffolds without conductive coating were mounted on the sticky conductive carbon tape (Ted Pella, Redding, California) on the top of aluminum stubs (Ted Pella, Redding, California) and examined under SEM with an accelerating voltage of 10 kV at low vacuum mode.

### Ethics statement

Animal usage complied with regulations set by the University of California, Los Angeles, Chancellor’s Animal Research Committee and was approved as animal protocol number 2005-169. All efforts were made to minimize pain and suffering. Two mice strains were used for these experiments: C57BL/6-Tg(Actb-EGFP)1Osb/J (“GFP”) (The Jackson Laboratory, Bar Harbor, ME) and wild type C57BL/6 (Charles River, Wilmington, MA).

### Feeder cells preparation

Embryonic fibroblasts, STO (SIM, Sandos Inbred Mouse) and MEF (C57BL/6) (both from ATCC) were expanded in STO medium (low glucose DMEM with GlutaMAX^TM^ supplemented with 10% FBS and 1x ABAM (All Invitrogen)) and suspended in STO medium with 10% DMSO for cryopreservation (~10^6^ cells/ml, 1 ml/vial). ~10^6^ feeder cells were thawed and cultured in gelatin coated T25 flasks with STO medium for a few days until confluency and treated with mitomycin C (Sigma). ~100 k cells were seeded onto gelatin-coated substrates such as 48-well plate (Coring Costar, Fisher Scientific), eight-chamber slide (Nunc™ Lab-Tek™ II; Thermo Scientific) and ePCL scaffold, and cultured at 37 °C with STO medium in a 10% CO_2_ incubator. Prepared feeder cells were used within 3 days.

### Primary intestinal smooth muscle cell mixture (ISMC Mix) isolation and culture

ISMC Mix were isolated from 7 to 10-day-old C57BL/6 neonates using previously described methods^12,38,40,50^. The intestines were removed via a midline incision, and smooth muscle strips, containing both longitudinal and circular muscle layers, were gently teased from the intestines using fine forceps and placed in Hank’s Balanced Salt Solution (HBSS) without calcium and magnesium (Invitrogen, Carlsbad, CA) with 1x antibiotic-antimycotic (ABAM) (Invitrogen) on ice. Then the smooth muscle strips were minced and incubated at 37 °C for 30~40 min in an enzyme solution containing collagenase (1.3 mg/ml Worthington Type II; Worthington Biochemical, Freehold, NJ), bovine serum albumin (BSA) (2 mg/ml), trypsin inhibitor (2 mg/ml), ATP (0.27 mg/ml) (all from Sigma, St. Louis, MO), and 10% HBSS with calcium and magnesium (Invitrogen) in HBSS without calcium and magnesium containing 1x ABAM. The tissue was washed twice with vigorous pipetting with 10% FBS in HBSS without calcium and magnesium containing 1x ABAM. As a preparation for immunomagnetic sorting (MACS), the resulting ISMC Mix was cultured in gelatin coated T25 flasks (ISMC Mix from ~4 pups in one T25 flask) for 3 days. Alternatively, 100 k ISMC Mix were seeded onto substrates (48 well plate, eight-chamber slide, ePCL scaffold) with STO cells or gelatin coating. ISMC Mix were cultured at 37 °C in a 5% CO_2_ incubator in FBS medium (Knockout™ D-MEM supplemented with 15% ECS-qualified FBS, 0.1 mM MEM Non-Essential Amino Acids solution, 2 mM L-glutamine, 0.1 mM 2-mercaptoethanol, 1x ABAM, and 10 mM HEPES (Invitrogen)). All ISMC Mix were cultured in FBS medium for the first 4 days followed by FBS or F12 medium (Advanced DMEM/F-12 supplemented with 2 mM L-glutamine, 1x ABAM, and 10 mM HEPES (Invitrogen)). For conditioned medium (CM) preparation, STO medium was replaced with FBS medium after the STO cells formed a confluent monolayer and treated with mitomycin C. The FBS medium was collected after 4~5 days of incubation, passed through a 0.2 μm pore size filter (Pall Corporation, Port Washington, NY), and mixed with fresh FBS medium (1:1 ratio) as CM. The medium was changed every 2~3 days.

### MOVAS cell culture

MOVAS cells (C57BL/6) (ATCC) were expanded in STO medium and suspended in STO medium with 10% DMSO for cryopreservation (~10^6^ cells/ml, 1 ml/vial). ~10^6^ feeder cells were thawed and cultured in gelatin coated T25 flasks with STO medium for a few days until confluency. ~100 k cells were seeded onto 48-well plate (Coring Costar, Fisher Scientific) with STO cells or gelatin coating in triplicates. All MOVAS cells were cultured in FBS medium for the first 4 days followed by FBS or F12 medium.

### Enrichment of ICC by immunomagnetic sorting (MACS)

After 3 days in culture, ISMC Mix were washed in PBS and incubated with 0.05% trypsin-EDTA (Invitrogen) at 37C for ~2 min The ICC marker, Kit, the epitope recognized by ACK2, is easily altered or masked during the enzymatic digestion process. Therefore, it is important to avoid over-digestion. The reaction was stopped by adding FBS medium. The dissociated cells were washed once with sorting buffer (1% BSA (Fisher Scientific) and 2 mM EDTA (Sigma) in PBS; ~pH7.2) and passed through a 100 µm cell strainer (Fisher Scientific). After centrifuging the cell suspension at 1000 rpm for 5 min, supernatant was aspirated completely. The cell pellet was re-suspended in sorting buffer and then well-mixed with the CD117 MicroBeads solution (Miltenyi Biotec, Germany; 1:5 in sorting buffer). The cell suspension was incubated for 15 minutes at 4 °C. For cells from four pups, 160 µL of sorting buffer and 40 µL of CD117 MicroBeads solution were used. Half the volume was used for cells isolated from two pups. The cell suspension was mixed with pipetting every 5 min. After being washed twice with the sorting buffer, the labeled cells were re-suspended in sorting buffer and 0.5 ml of the cell suspension was passed through pre-wetted MS magnetic columns placed in a strong magnet (MiniMACS; Miltenyi Biotec) at a time. Cells from four pups were re-suspended in 4 ml sorting buffer and passed through two MS columns, 2 ml per each. After passing all of the cell suspension, MS columns were washed once with 1 ml of sorting buffer. Unlabeled cells were collected as flow-through (MACS− cells), while labeled cells retained in the columns were flushed out with 1 ml of sorting buffer after removing the column from the magnet (MACS+ cells). MACS+ cells were seeded onto gelatin or feeder cells and cultured at 37 °C, 10% CO_2_ incubator in FBS medium.

For P-MACS culture, the resultant MACS+ cells from 4 pups were all cultured on a STO-seeded well of a 6-well plate for 7 days, passaged with MACS, and subsequently cultured on STO cells in FBS medium. The medium was changed every 2~3 days.

For MACS0, MACS− and MACS−/+ culture, CD117 MicroBeads labeled cells without passing through the MS magnetic column (MACS0) and MACS− cells were cultured on STO cells in FBS medium for 4 days followed by F12 medium. We also added MACS+ cells back to MACS− cell culture 5 days after they were cultured on STO cells in order to recover its spontaneous contractility (MACS−/+). The MACS−/+ cells were cultured in FBS medium for 7 days, followed by F12 medium (2 days after adding the MACS+ cells onto MACS− cells). The medium was changed every 2~3 days.

### Measurement of pacemaker activity in purified ICC (MACS+ cells)

To monitor oscillations in intracellular Ca^2+^ concentration, cultured ICC (MACS+ cells) were incubated with 1 part of 2X Fluo-4 Direct™ calcium reagent loading solution without probenecid (Invitrogen) and 1 part of FBS medium at 37 °C, 5% CO_2_ incubator for 0.5~1 hour. Incubating for too short of a time will result in a weak signal while too long will result in an increased number of floating dead cells. Fluorescent images (680 × 512 pixels) were acquired with an Olympus IX71 microscope using cellSens software (Olympus, Center Valley, PA) (acquisition rate: 4.8~7.7 Hz; excitation wavelength: 494 nm; emission wavelength: 516 nm), and analyzed using custom MATLAB programs^12^ (region of interest analysis, Supplementary Fig. 6; fast-forward and gray scale normalization, Supplementary Fig. 7). In order to keep the temperature close to 37 °C^51,52^, cells were limited to 5 min at room temperature for image acquisition and were returned to the incubator before repeating the process.

### Colonoscopic injection of MACS+ cells cultured on STO cells

Eight to 12 week old male and female C57BL/6 J mice (n = 3) were placed on water only diet overnight. Anesthesia was induced with 3% isoflurane then decreased to 1% for maintenance. Colonoscopy was performed by introducing a lubricated 1.9 mm endoscope (Karl Storz, Tuttlingen, Germany) into the mouse rectum, as previously described^53^. GFP + MACS+ cells were cultured on STO cells for 4 days and MACS purified prior to the colonoscopic injection. The resulting P-MACS+ cells were re-suspended in low glucose DMEM with GlutaMAX^TM^ (Invitrogen) to a concentration of 5 × 10^5^ cells/ml and mixed with 1.5% permanent carbon ink (Spot; GI Supply, Camp Hill, PA) for later identification of the injection sites. Using a 30 gauge custom-made needle fitted with pulled and beveled capillary tubing^53^, 100 µL of the cell suspension was injected into the submucosa of the ventral rectum (n = 12 total). Mice were monitored for appropriate recovery from anesthesia and placed back in standard laboratory mouse cages with full access to water and chow. All mice were sacrificed 7 days after the cell injection procedure and colon specimens were isolated.

### Histology and immunofluorescence staining

Samples, including cell-seeded ePCL and P-MACS+ injected intestine, were formalin-fixed and processed for paraffin embedding. Serial 5 µm sections were cut and adhered to glass slides. Unstained slides were prepared for immunofluorescence staining. Slides were de-waxed with xylene and rehydrated with serial dilutions of ethanol. Next, slides were incubated in a citric buffer (Biogenex, San Ramon, CA) for 15 min at 95–100 °C and allowed to cool for 15 minutes in an over flowing water bath. *In vitro* cultures and native smooth muscle strips were fixed without histologic processing. For Kit and Ano1 staining, samples were fixed with acetone for 30 min at 4 °C. For all the other staining, samples were fixed with 10% formalin for 30 min at room temperature. After three washes with PBS, samples were treated with a blocking buffer containing 5% normal goat serum (Vector Labs, Burlingame, CA), 0.1% Triton X-100 (Sigma) in PBS for 1 hour at 4 °C. Samples were then incubated with the following primary antibodies: Kit (1:500; eBioscience: 16-1172-82), Ano1 (1:500; Abcam: ab53212), MHC (1:100; Abcam: ab53219), β-tubulin (1:500; Abcam: ab78078), GFP (1:500; Aves: GFP-1020), and SCF (1:500; ab64677) diluted in staining buffer containing 5% normal goat serum in PBS overnight at 4 °C. After two washes in PBS, samples were incubated in the dark with their corresponding Alexa Fluor® secondary antibodies (1:500; Invitrogen) and Alexa Fluor® 594 Palloidin (1:100; Invitrogen) diluted in staining buffer for 2~3 hours at room temperature. After three washes with PBS, Prolong Gold with DAPI (Invitrogen) was applied to each section and the slides were covered with glass coverslips. The slides were allowed to cure at 4 °C before storing at −80 °C. Fluorescent images were acquired with an Olympus IX71 microscope with cellSens software (Olympus, Center Valley, PA) or confocal laser microscopy (TCS SP2 AOBS, TCS SP2 MP AOBS; Leica).

### DNA/RNA extraction and qPCR

DNA and RNA were extracted and purified from *in vitro* cultures using the DNeasy and RNeasy kits (Qiagen, Valencia, CA), respectively following the manufacture’s protocol. RNA samples were cleaned with RNase-Free DNase set (Qiagen) during the procedure. The qPCR reactions were prepared using a PCR master mix and the Quantitect Probe RT-PCR Kit (Qiagen) and performed on an ABI 7500 Real Time PCR System (Applied Biosystems). The primers and probe for *gfp*-DNA and *Kit*-RNA were custom- designed and purchased from Eurofins MWG Operon, (Huntsville, AL): *gfp*-fw, 5′-ACTACAACAGCCACAACGTCTATATCA-3′; *gfp*-rev, 5′-GGCGGATCTTGAAGTTCACC-3′; *gfp*-pb, and 5′-[6-FAM]CCGACAAGCAGAAGAACGGCATCA[TAMRA-Q]-3′; *Kit*-fw, 5′-CCGTGAACTCCATGTGGCTAAAGA; *Kit*-rev, 5′-GGTGCCAGCTATTGTGCTTTACCT-3′; *Kit*-pb, 5′-[6-FAM]TGAACCCTCAGCCTCAGCACATAGC[TAMRA-Q]-3′. All the other primer/probe mixtures were purchased from Applied Biosystems: *Myh11* (Mm00443013_m1), *Tubb3* (Mm00727586_s1), *Kitl* (Mm00442972_m1), *Acta2* (Mm01546133_m1), *Ano1* (Mm00724407_m1) and *Gapdh* (Mm99999915_g1). DNA qPCR results were analyzed using a standard prepared with known number cells from ISMC Mix. RNA qPCR results were analyzed using *Gapdh* as the housekeeping gene and de-epithelialized intestine from 7 to 10-day-old C57BL/6 neonates as reference. For MOVAS cells, 100 k cells from day 0 were used as reference.

### MTS assay

MTS assay was conducted to test the viability of mitomycin C treated STO cells over time. 100 k mitomycin C treated STO cells were seeded on to 48-well plate in STO medium overnight, followed by the same series of medium as the preparation for ISMC Mix feeder layer. At each time point, wells with cells and without cells (control) were incubated with a mixture of CellTiter 96® AQueous One Solution Reagent (Promega) in appropriate medium (1:5 volume ratio) at 37 °C in a 5% CO_2_ incubator for an hour. The absorbance was measured at 490 nm (Synergy^TM^ HT; BioTek). Data were normalized to controls.

### Data Analysis

The frequency of spontaneous contractions of the cells was analyzed manually from recorded videos (680 × 512 pixels; acquisition rate: 5.0 Hz) acquired with an Olympus IX71 microscope using cellSens software (Olympus, Center Valley, PA). The area, height and width of the most contracted ePCL scaffolds were normalized to that of the most relaxed ePCL scaffolds with ImageJ using images extracted from a representative video and manually outlined ePCL scaffolds. In order to compare the size of feeder cells, area of MEF was normalized to that of STO cells with ImageJ. For the cell alignment quantification, ImageJ was used to conduct circularity analysis (DAPI) and the plugin OrientationJ was used to conduct coherency analysis (Kit, MHC, GFP and β-tubulin).

### Statistical analysis

One-way ANOVA followed by Tukey test or two-tailed unpaired Student’s t-test was used depending on the number of simultaneous conditions to analyze. Levels of significance were set at ***P < 0.0001, **P < 0.01, and *P < 0.05. Data were expressed as the mean ± the standard deviation (s.d.). n indicates the number of independent experiments (different primary cell isolations from different animals) except for the following cases: Fig. 2d,e, n = number of cell cultures (from 3 different independent experiments); Fig. 3c, n = number of images (from one independent experiment); Fig. 4c, n = number of videos (from two independent experiments); Fig. 5d,e, n = number of images (extracted from a representative video).

## Electronic supplementary material

Supplementary figures

Video 1

Video 2

Video 3

Video 4
