# Supplementary material for: Bioengineering functional smooth muscle with spontaneous rhythmic contraction in vitro
Source: Sci Rep. 2018 Sep 10;8:13544. doi: 10.1038/s41598-018-31992-4 (PMC6131399; doi:10.1038/s41598-018-31992-4)
Supplement: Supplementary file 1 — Supplementary figures [file 41598_2018_31992_MOESM1_ESM.docx]

**Bioengineering functional smooth muscle with spontaneous rhythmic contraction *in vitro***

Masae Kobayashi ^1^, Hassan A. Khalil ^2^, Nan Ye Lei ^1, 2^, Qianqian Wang ^1^, Ke Wang ^3^, Benjamin M. Wu ^1, 4^, James C.Y. Dunn ^1, 2, 5^

^1^ Department of Bioengineering, Henry Samueli School of Engineering, University of California, Los Angeles, Los Angeles, CA 90095, USA

^2^ Department of Surgery, David Geffen School of Medicine at UCLA, University of California, Los Angeles, Los Angeles, CA 90095, USA

^3^ Department of Computer Science, University of North Carolina Chapel Hill, North Carolina , NC 27514, USA

^4^ Division of Advanced Prosthodontics & Weintraub Center for Reconstructive Biotechnology, University of California, Los Angeles, Los Angeles, CA 90095, USA

^5^ Department of Surgery, Stanford University School of Medicine, Stanford, CA 94305, USA

^*^ Correspondence should be addressed to

James Dunn (jdunn2@stanford.edu)

Alway M116

300 Pasteur Drive

Stanford, CA 94305


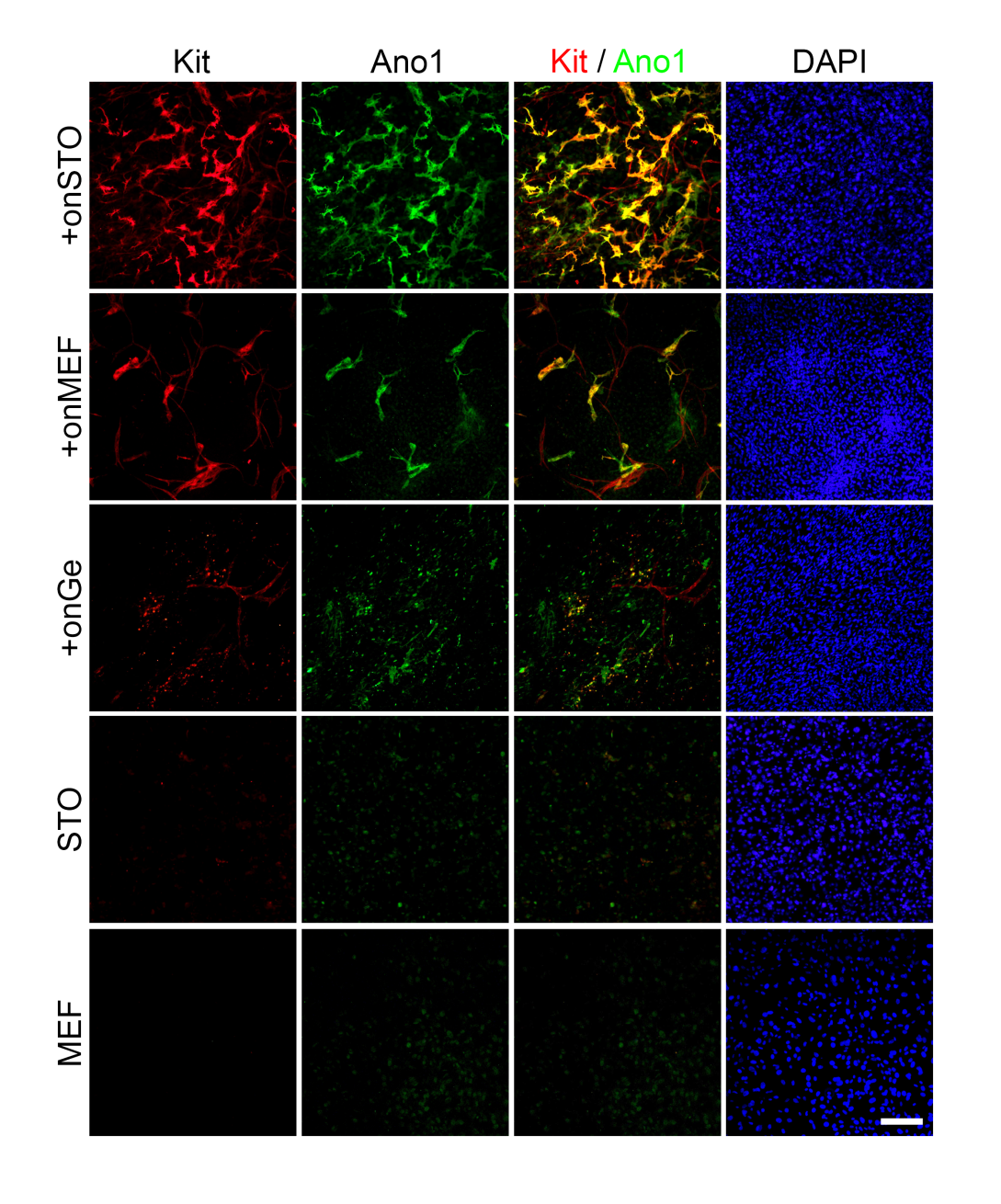


**Supplementary Figure 1** | Comparison of MACS+ cells cultured on STO cells, MEF cells, and gelatin *in vitro*. Immunofluorescence of ICC markers: Kit (red) and Ano1 (green) and with co-localization (yellow), and nuclear marker DAPI (blue). 60k MACS+ cells on different substrates and feeder cells alone (STO, MEF: controls) were cultured for 7 days. Scale bar, 200 µm. STO = Mouse Embryonic Fibroblast (Santos Inbred Mouse, SIM). MEF = Mouse Embryonic Fibroblast (C57BL/6). Ge = gelatin coating. Feeder cells (STO, MEF) were mitomycin C treated.


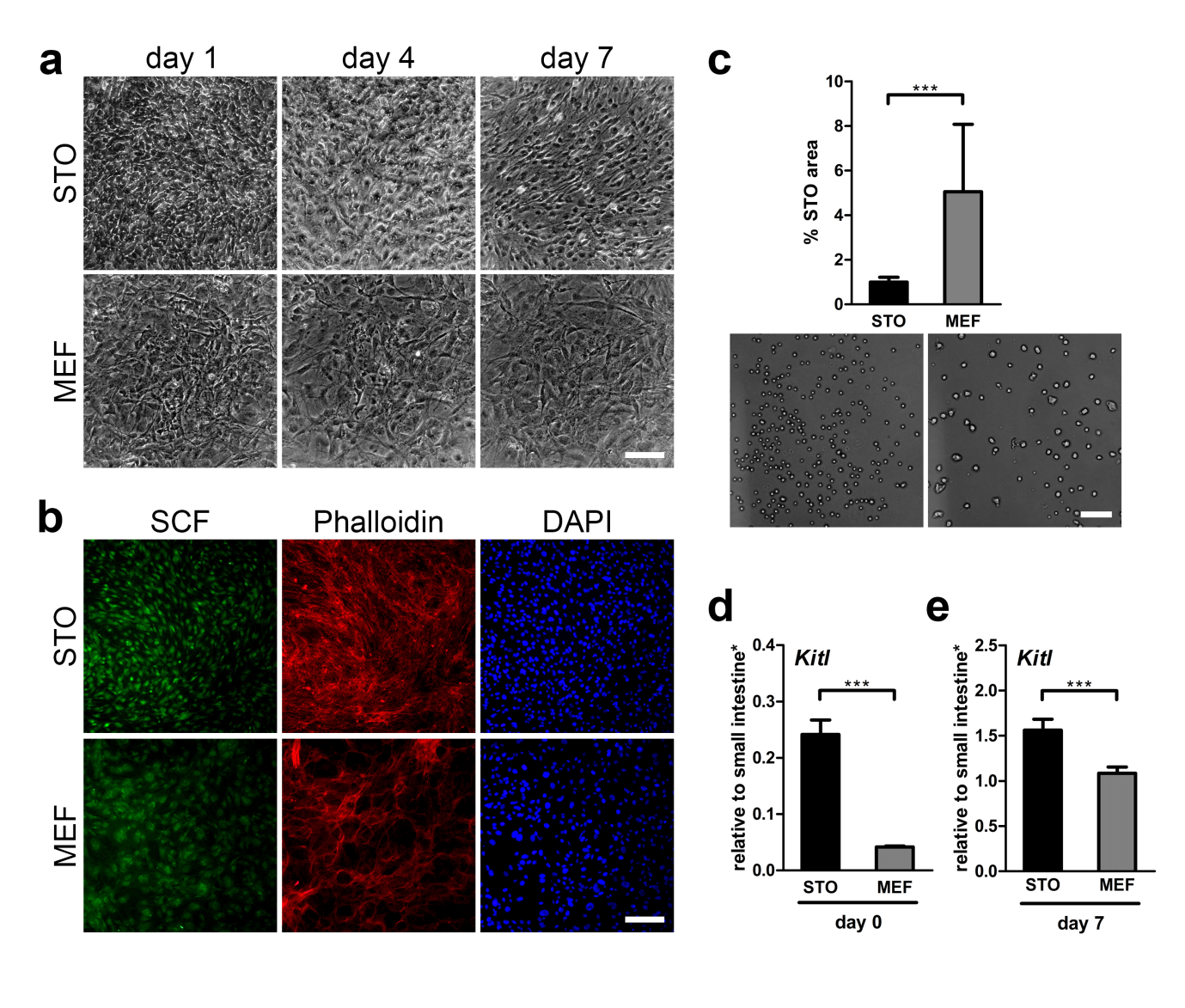


**Supplementary Figure 2** | Comparison of STO and MEF feeder cells cultured *in vitro* over time. 100k feeder cells were seeded on gelatin in STO medium overnight and cultured in FBS medium for 7 days. (**a**) Phase contrast images of the feeder cells. Scale bar, 100 µm. (**b**) Immunofluorescence of feeder cells with Kit ligand marker SCF (green), cytoskeleton marker phalloidin (red), and nuclear marker DAPI (blue). Scale bar, 200 µm. (**c**) Comparison of the size of feeder cells suspended in medium at day 0 before seeding. Phase contrast images were taken for cell area comparison. Area of MEF cells were normalized to that of STO cells (*n=70* cells; **** P* < 0.0001). Scale bar, 100 µm. (**d-e**) Feeder cells were compared for their *kitl* mRNA expression at day 0 as a cell suspension (**d**: *n=3*; triplicate samples) and at day 7 of culture (**e**: *n=4*; quadruplicate samples). **** P* < 0.001. FBS = 15% FBS in DMEM. *Samples were normalized to de-epithelialized intestine. Error bars, s.d.


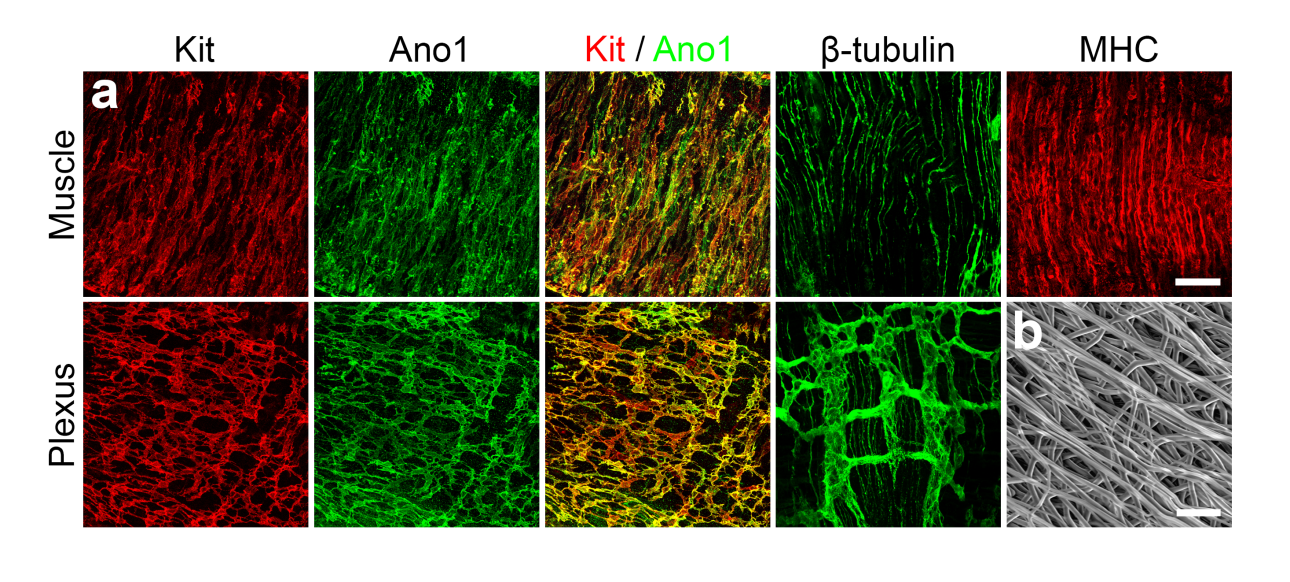


**Supplementary Figure 3** | Cell alignment in the native murine small intestine. (**a**) Confocal images of immunofluorescent murine muscle strips with ICC markers showing co-localization (yellow) of Kit (red) and Ano1 (green), neuronal marker β-tubulin (green) and SMCs marker MHC (red). Scale bar, 50 µm. (**b**) Scanning electron micrographs of ePCL scaffold. The fiber alignment of ePCL scaffold was used to induce cell alignment. Scale bar, 25 µm. Muscle = muscle layer of murine small intestine. Plexus = myenteric plexus of murine small intestine.


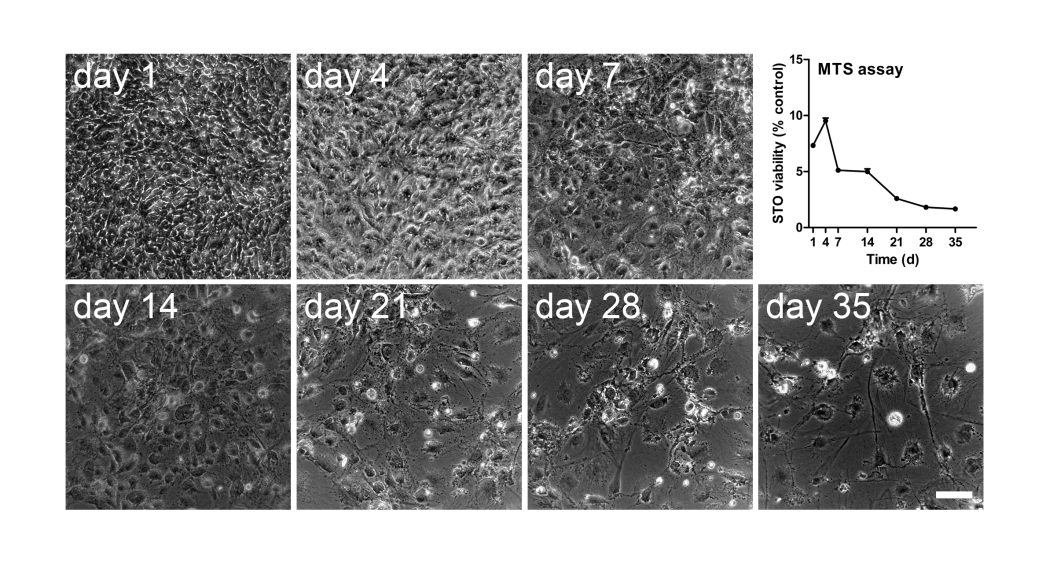


**Supplementary Figure 4** | Viability of mitomycin C treated STO cells *in vitro* over time. 100k STO cells were cultured under the same conditions as with ISMC Mix co-culture. Cells were seeded on gelatin in STO medium overnight and cultured in FBS medium for 4 days followed by F12 medium. Phase contrast images and MTS assay measurements (*n=3*; triplicate samples; error bars, s.d.) were taken at each time point. Scale bar, 100 µm. F12 = advanced DMEM/F12.


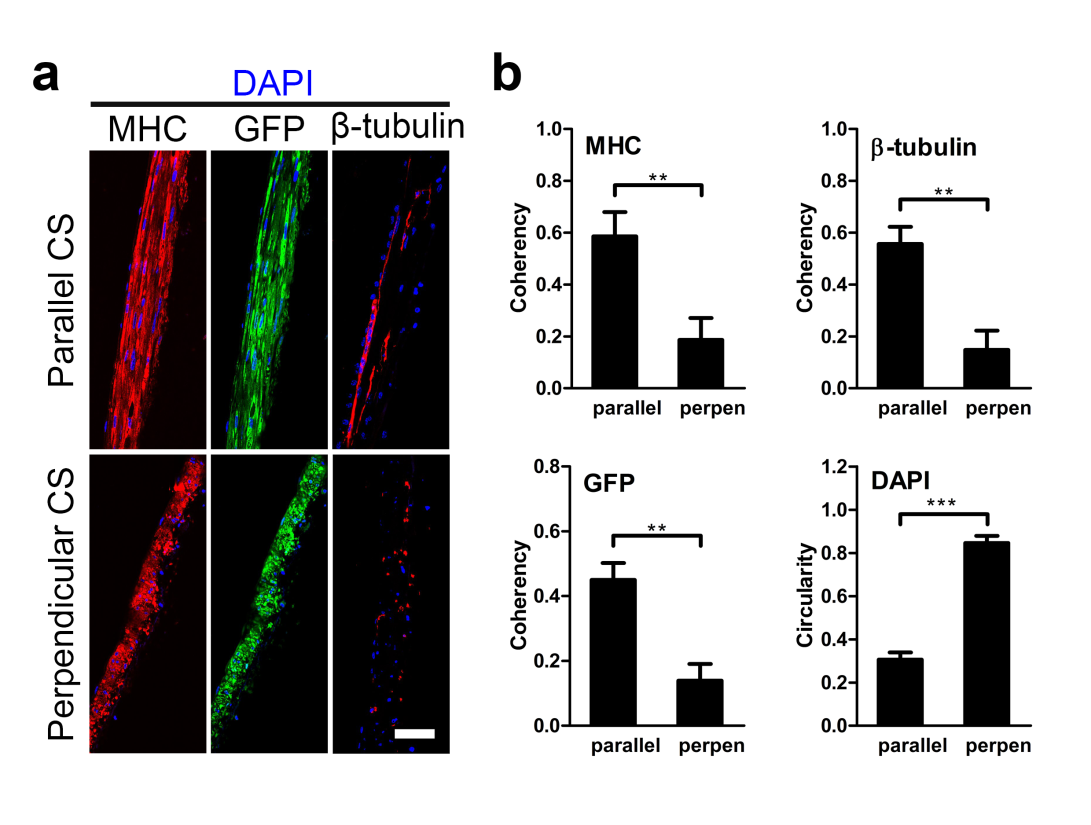


**Supplementary Figure 5** | Alignment quantification of infiltrated ISMC Mix inside ePCL scaffolds at 10 weeks. Non-sorted cells from enzymatically digested intestinal muscle strips, ISMC Mix, were cultured on STO-seeded ePCL scaffolds. 100k ISMC Mix were cultured in FBS medium for the first 4 days before changing to F12 medium. ePCL scaffolds were cut in two orthogonal planes to expose cross section (CS) parallel or perpendicular (perpen) to ePCL’s aligned fibers. (**a**) Confocal images of immunostained ISMC Mix with SMCs marker MHC (red), GFP (green), neuronal marker β-tubulin (red), and nuclear marker DAPI (blue) in parallel and perpendicular cross sections. Scale bar, 50 µm. (**b**) Coherency analysis (MHC, GFP and β-tubulin) and circularity analysis (DAPI) of infiltrated ISMC Mix inside ePCL scaffolds (*n = 3*). Coherency analysis = range from 0 (perfect random) to 1 (perfect alignment) indicating cell alignment. Circularity analysis = range from 0 (most elongated shape) to 1 (perfect circle) indicating elongation of nuclei due to cell alignment. *Samples were normalized to de-epithelialized intestine. Error bars, s.d. **** P* < 0.0001, *** P* < 0.01.

function multiROI()

[filename,filepath,~] = uigetfile('*.*', 'All Files (*.*)');

video_handle = VideoReader(fullfile(filepath, filename));

reference_frame = read(video_handle, 1);

frame_num = video_handle.NumberOfFrames;

% compute an average intensity change map

average_intensity_change = zeros(video_handle.Height, video_handle.Width);

for frame_idx = 1 : frame_num

curr_frame = read(video_handle, frame_idx);

average_intensity_change = average_intensity_change + im2double(curr_frame(:, :, 2));

end

average_intensity_change = average_intensity_change ./ frame_num;

%figure(1), subplot(121); imshow(reference_frame);

figure(1), imagesc(average_intensity_change); axis image;

roi_num =input('Input the number of ROIs you want to select in this video:');

roi_masks = cell(roi_num, 1);

roi_images = cell(roi_num, 1);

for roi_idx = 1 : roi_num

message = sprintf('%d out of %d ROI. Left click and hold to begin drawing.\nSimply lift the mouse button to finish',...

roi_idx, roi_num);

uiwait(msgbox(message));

hFH = imfreehand();

roi_masks{roi_idx} = hFH.createMask();

pos = hFH.getPosition();

% define bounding box

x1 = max(1, min(round(min(pos(:,2))), video_handle.Height));

y1 = max(1, min(round(min(pos(:,1))), video_handle.Width));

x2 = max(1, min(round(max(pos(:,2))), video_handle.Height));

y2 = max(1, min(round(max(pos(:,1))), video_handle.Width));

roi_image_r = reference_frame(:, :, 1) .* uint8(roi_masks{roi_idx});

roi_image_g = reference_frame(:, :, 2) .* uint8(roi_masks{roi_idx});

roi_image_b = reference_frame(:, :, 3) .* uint8(roi_masks{roi_idx});

roi_images{roi_idx} = cat(3, ...

roi_image_r(x1:x2, y1:y2), ...

roi_image_g(x1:x2, y1:y2), ...

roi_image_b(x1:x2, y1:y2));

end

fprintf('Start processing.\n');

average_intensity = zeros(frame_num, roi_num);

average_interval = 1.0 / video_handle.FrameRate;

for i = 1 : frame_num

raw_frame = read(video_handle, i);

green_channel = double(raw_frame(:, :, 2));

% abandon red and blue channel, because mostly no data is available

for roi_idx = 1 : roi_num

average_intensity(i, roi_idx) = mean(green_channel(roi_masks{roi_idx}));

end

end

% filter out the first dark period

thres = mean(average_intensity) - 3 * std(average_intensity);

valid_frame_indices = zeros(roi_num, 1);

for roi_idx = 1 : roi_num

indices = sort(find(average_intensity(:, roi_idx) >= thres(roi_idx)), 'ascend');

valid_frame_indices(roi_idx) = indices(1);

end

for roi_idx = 1 : roi_num

figure; hold on;

title(sprintf('ROI %d', roi_idx));

subplot(231); imagesc(roi_images{roi_idx}(:, :, 2)); axis image; title(sprintf('Image ROI %d', roi_idx));

axis off;

truncated_average_intensity = average_intensity(valid_frame_indices(roi_idx):end, roi_idx) %; want to know this number

truncated_timestamps = [valid_frame_indices(roi_idx) : frame_num] .* average_interval;

truncated_timestamps'

Y = fft(truncated_average_intensity);

n=length(Y);

Y=Y(1:ceil(n/2));

n=length(Y);

mY=abs(Y);

subplot(233); hold on;

FREQ=(0:n-1)*(video_handle.FrameRate /(2*n));

semilogy(FREQ, mY);

xlabel('Frequency (Hz)');

title('Periodogram of Depolarization');

subplot(232); hold on;

xlabel('Time (seconds)');

ylabel('Intensity (percent)');

title('Mean Intensity Over Time');

plot(truncated_timestamps, truncated_average_intensity);

% Establish characteristics of the FFT (Periodogram) of the intensity plot

% of depolarizing cells. This plot is the top right image.

% I also create a zero-centered Periodogram for easier visualization. This

% plot is on the bottom left figure.

Z=fftshift(mY);

f0 = (-n/2:n/2-1)*.5*(video_handle.FrameRate/(length(Y))); % 0-centered frequency range

subplot(235); hold on;

plot(f0,Z);

xlabel('Frequency (Hz)');

title('Zero-shift Periodogram of Depolarization');

[pks,locs] = findpeaks(Z);

[pkvals,idx] = sort(pks,'descend'); %sort to vector

pkvals(2); %second largest value - the first is always 0 and doesn't mean anything

index=find(Z==pkvals(2));

mainFrequencyStr=num2str(f0(index));

plot(f0(index),Z(index),'r.', 'MarkerSize',25);

text(f0(index),Z(index),['Frequency = ',mainFrequencyStr, ' Hz']);

Frequency = f0(index);

axis([0 ceil(10*f0(index)) 0 ceil(2*pkvals(2))]);

fprintf('ROI %d: Frequency %f Hz\n', roi_idx, f0(index));

% I do the same thing for the inverse of the frequency, aka the period.

period=1./FREQ;

subplot(236); hold on;

plot(period,mY);

[pks2,locs2] = findpeaks(mY);

[pkvals2,idx2] = sort(pks2,'descend'); %sort to vector

pkvals2(1); % largest value

index2=find(mY==pkvals2(1));

mainPeriodStr=num2str(period(index2));

plot(period(index2),mY(index2),'r.', 'MarkerSize',25);

text(period(index2),mY(index2),['Period = ',mainPeriodStr, ' Seconds']);

xlabel('Period (seconds)');

axis([0 ceil(2*period(index2)) 0 ceil(2*pkvals2(1))]);

fprintf('ROI %d: Period %f seconds.\n', roi_idx, period(index2));

end

end

**Supplementary Figure 6** | Matlab code for analyzing Ca^2+^ oscillation videos. Multiple regions of interest (ROIs) can be selected and the average fluorescent intensity within the selected regions is calculated for each frame. Outputs include time-course change in fluorescence intensity, frequency and period for each selected ROI.

%Reset everything

clear all;

close all;

bordervector = [0 16 30 34 48 64];

colorscale=zeros(64,3);

for j=1:64

if bordervector(1)<=j && j<=bordervector(2)

colorscale(j,1)=0;

colorscale(j,2)=0;

colorscale(j,3)=1;

end

if bordervector(2)<j && j<=bordervector(3)

scale1=((j-15)*18)/270;

colorscale(j,1)=scale1;

colorscale(j,2)=scale1;

colorscale(j,3)=1;

end

if bordervector(3)<j && j<=bordervector(4)

colorscale(j,:)=1;

end

if bordervector(4)<j && j<=bordervector(5)

scale2=(255-((j-34)*18))/255;

colorscale(j,1)=1;

colorscale(j,2)=scale2;

colorscale(j,3)=scale2;

end

if bordervector(5)<j && j<=bordervector(6)

colorscale(j,1)=1;

colorscale(j,2)=0;

colorscale(j,3)=0;

end

end

% Note: a large portion of this code was directly taken and modified

% from code used in "Smooth muscle strips for intestinal tissue engineering"

% by Chris Walthers.

%Ask user to choose a file to run. This should be a .wmv file. If it is

%not, you can easily convert with Windows Live Movie Maker

[FileName,PathName,filterindex] = uigetfile('*.*', 'All Files (*.*)');

%The following allows you to shorten the video produced by this code.

framenum=input('How many frames do you want to skip between each frame captured? (Must be greater than 0) ');

fps=30; %frames per second; wmvs are always 30

sample=fps/(framenum); %establish how many frames per second new video will have, important for the FFT part

% Saves the new video in the same folder as the input video

outputFolder = PathName;

% Read in the movie.

mov = VideoReader(FileName);

new_FileName = strrep(FileName, '.wmv', '_CAcode_output');

% Determine how many frames there are, and pixels, and final number of

% frames

nFrames = mov.NumberOfFrames;

videoHeight = mov.Height;

videoWidth = mov.Width;

newframes=floor(nFrames/(framenum+1));

% Preallocate movie structure.

movie1(1:newframes) = struct('cdata', zeros(videoHeight, videoWidth, 3, 'uint8'), 'colormap', []);

% % Read one frame at a time.

for k = 1 : newframes

movie1(k).cdata = read(mov, k*(framenum+1)-framenum+1);

end

%Preallocate structure for subsequent structures

greendata=zeros(videoHeight,videoWidth,newframes);

subtracted=zeros(videoHeight,videoWidth,(newframes-1));

for l=1:newframes

greendata(:,:,l)=movie1(l).cdata(:,:,2);

end

% Display the freehand mask in a 6 panel window with some cool extra figures

figure('units','normalized','outerposition',[0 0 1 1]);

workspace; % Make sure the workspace panel is showing.

fontSize = 16;

% colormap(colorscale);

% colormap jet;

colormap gray;

colormap(flipud(colormap));

% colormap(flipud(colormap));

%Do some housekeeping

clear movie1;

% Prepare the new video file.

% New file will have same file name as before, but with _CAcodeFFT.avi at

% the end.

% vidObj = VideoWriter(new_FileName,'Uncompressed AVI');

vidObj = VideoWriter(new_FileName,'Motion JPEG AVI');

open(vidObj);

% This is a "for loop" that subtracts the first image in the video from all

% subsequent images. In effect, this "normalizes" every frame of the video

% to the first frame (arbitrarily... you could do the same with any frame

% from the video for similar effect). Now the new video will show changes

% in intensity from the first frame.

% colormap(colorscale);

for m=2:newframes

subtracted(:,:,m)=imsubtract(greendata(:,:,m), greendata(:,:,3));

image((subtracted(:,:,m))+31);

currFrame=getframe;

writeVideo(vidObj,currFrame);

end

% This line makes it easier to view stills from your video exactly as they

% appear in the video

subtracted=subtracted+31;

% % % This code plays a series of stills for reviewing your video after

% making it

% % selectImage = implay(subtracted);

% % selectImage.Visual.ColorMap.UserRangeMin=0;

% % selectImage.Visual.ColorMap.UserRangeMax=64;

% % selectImage.Visual.ColorMap.UserRange = 1;

% % set(findall(0,'tag','spcui_scope_framework'),'position',[100 150 (videoWidth)*1.1 (videoHeight)*1.1]);

% Close the file.

close(vidObj);

% Housekeeping

% clear greendata; clear subtracted;

**Supplementary Figure 7** | Matlab code for processing Ca^2+^ oscillation videos. Original video can be condensed by capturing only every nth frame (every 3th frame was used to produce supplementary video 1). The intensity values from the first frame in the video are subtracted as baseline intensity from each subsequent frame to create a normalized intensity profile compared to the first frame. Changes in fluorescent intensity, corresponding to calcium concentration, are color-mapped to show in grayscale with an increase in black, decrease in white, or no change in gray relative to the first frame.

**Supplementary Video 1** | Ca^2+^ oscillation of ICC in the culture of MACS+ cells on STO at day 7. The original video was processed with matlab so that it is about three times faster than the real time and color normalized to the first frame of the video, where higher intensity shows up lighter and lower intensity shows up darker.

**Supplementary Video 2, 3** | ISMC Mix without GFP expression (**2**) and with GFP expression (**3**) cultured on STO cells *in vitro* for a week.

**Supplementary Video 4** | ISMC Mix were cultured on STO cells seeded ePCL scaffolds for 8 weeks.
